# Airborne copper exposure in school environments associated with poorer motor performance and altered basal ganglia

**DOI:** 10.1002/brb3.467

**Published:** 2016-04-22

**Authors:** Jesus Pujol, Raquel Fenoll, Dídac Macià, Gerard Martínez‐Vilavella, Mar Alvarez‐Pedrerol, Ioar Rivas, Joan Forns, Joan Deus, Laura Blanco‐Hinojo, Xavier Querol, Jordi Sunyer

**Affiliations:** ^1^ MRI Research UnitHospital del MarBarcelonaSpain; ^2^ Centro Investigación Biomédica en Red de Salud Mental, CIBERSAM G21BarcelonaSpain; ^3^ Centre for Research in Environmental Epidemiology (CREAL)BarcelonaCataloniaSpain; ^4^ Pompeu Fabra UniversityBarcelonaCataloniaSpain; ^5^ Ciber on Epidemiology and Public Health (CIBERESP)BarcelonaSpain; ^6^ Institute of Environmental Assessment and Water Research (IDAEA‐CSIC)BarcelonaCataloniaSpain; ^7^ Department of Clinical and Health PsychologyAutonomous University of BarcelonaBarcelonaSpain; ^8^ IMIM (Hospital del Mar Medical Research Institute)BarcelonaCataloniaSpain

**Keywords:** Air pollution, brain development, copper, diffusion tensor imaging, fMRI, neurodegenerative disorders

## Abstract

**Introduction:**

Children are more vulnerable to the effects of environmental elements. A variety of air pollutants are among the identified factors causing neural damage at toxic concentrations. It is not obvious, however, to what extent the tolerated high levels of air pollutants are able to alter brain development. We have specifically investigated the neurotoxic effects of airborne copper exposure in school environments.

**Methods:**

Speed and consistency of motor response were assessed in 2836 children aged from 8 to 12 years. Anatomical MRI, diffusion tensor imaging, and functional MRI were used to directly test the brain repercussions in a subgroup of 263 children.

**Results:**

Higher copper exposure was associated with poorer motor performance and altered structure of the basal ganglia. Specifically, the architecture of the caudate nucleus region was less complete in terms of both tissue composition and neural track water diffusion. Functional MRI consistently showed a reciprocal connectivity reduction between the caudate nucleus and the frontal cortex**.**

**Conclusions:**

The results establish an association between environmental copper exposure in children and alterations of basal ganglia structure and function.

## Introduction

Brain development is extraordinarily complex and extends into adulthood to achieve maximum effectiveness in cognition and skills (Pujol et al. 1993; Paus 2005). Nonetheless, both the complexity and duration of such a process expose developing children to the potentially harmful effects of environmental elements. A variety of air pollutants are among the identified factors causing neural damage at toxic concentrations (Grandjean and Landrigan 2014). It is not obvious, however, to what extent the tolerated high levels of urban air pollutants are able to generate subtle, subclinical alterations in the developing brain in school‐age children.

Copper is an air pollutant that may potentially interfere with brain development. Although this element is necessary for cellular metabolism, abnormal copper levels lead to relevant brain impairment (Scheiber et al. 2014). The deleterious effect of an excess of copper is well‐known from Wilson's disease, an inherited metabolic disorder affecting the basal ganglia with symptoms usually evident from preadolescence onward and mainly in the form of deficient motor control (Bandmann et al. 2015).

We have tested the potential harmful effects of copper as an air pollutant in children aged from 8 to 12 years using specific motor testing and brain structural and functional imaging. Exposure to airborne copper at school was estimated for 2836 children in the city of Barcelona. Both the speed and consistency of motor response were measured to test the integrity of motor function in the whole sample. High‐resolution 3D MRI, DTI (diffusion tensor imaging), and functional MRI were used to directly test the potential repercussion on brain anatomy, architecture, and function in a subgroup of 263 children.

## Methods

### Participants

This study was developed in the context of the BREATHE project (The European Commission: FP7‐ERC‐2010‐AdG, ID 268479) aimed at assessing the impact of long‐term exposure to traffic‐related air pollutants on school children. A total of 2897 children participated in the whole survey from 39 representative schools of the city of Barcelona (Sunyer et al. 2015). A group of 2836 children completed the behavioral testing (mean age at the study end, 9.4 years; SD 0.9; and range 7.9–12.1 years) and served to test the effect of copper on motor function. From this sample, 1564 families were invited to participate in the MRI study via post, e‐mail, or telephone, and 810 of them gave an initial positive response. The recruitment of this group was consecutive with the aim of including children from all participating schools. Parents of 491 children were directly contacted. Consent to participate was finally not obtained in 165 cases, 27 children were lost before the assessment and 21 children were not eligible because of dental braces. The finally selected MRI group included 278 participants, 263 of whom completed the imaging protocol (mean age of 9.7 years, SD 0.9 and range, 8.0–12.1 years) and served to test direct effects of copper on brain. Study design and participant selection is fully described in Sunyer et al. (2015). Table 1 reports the characteristics of the study samples.

**Table 1:** Characteristics of study samples

	Whole sample (*n* = 2836)	MRI sample (*n* = 263)
Gender	49.5% girls 50.5% boys	48.3% girls 51.7% boys
Age, years, mean ± SD (range)	9.4 ± 0.9 (7.9–12.1)	9.7 ± 0.9 (8.0–12.1)
Overall school achievement, 5‐point scale	3.5 ± 1.1 (1–5)	3.7 ± 1.0 (1–5)
Difficulties score (SDQ), range 0–40	8.4 ± 5.2 (0–32)	8.8 ± 5.3 (0–25)
Obesity: Normal	72.1%	71.4%
Overweight, BMI ≥ 25–29.9 kg/m^2^	18.5%	18.4%
Obesity, BMI ≥ 30 kg/m^2^	9.4%	10.2%
Mother education (5‐point scale, 5 = University)	4.4 ± 0.8 (1–5)	4.5 ± 0.8 (1–5)
Father education (5‐point scale, 5 = University)	4.4 ± 0.8 (1–5)	4.4 ± 0.8 (1–5)
Vulnerability index^a^–Home	0.45 ± 0.21 (0.06–1.0)	0.43 ± 0.21 (0.06–0.90)
Vulnerability index^a^–School	0.42 ± 0.21 (0.13–0.84)	0.43 ± 0.22 (0.13–0.84)
Public/Nonpublic school	36% vs. 64%	43% vs. 57%
Task performance		
Reaction time (mean of medians, msec)	671.5 ± 124.6 (389–1277)	650.6 ± 119.9 (431–1091)
Reaction time standard deviation (msec)	235.7 ± 91.1 (60.6–598.6)	222.9 ± 91.2 (77.5–571.6)
Commission errors (number)	4.0 (3.1%) ± 4.2 (0–50)	4.3 (3.4%) ± 5–0 (0–49)
Omission errors (number)	1.4 (1.1%) ± 3.6 (0–94)	1.6 (1.3%) ± 3.9 (0–44)

BMI, body mass index; SDQ, Strengths and Difficulties Questionnaire.

a Neighborhood socioeconomic status vulnerability index based on the level of education, unemployment, and occupation at the census tract (Atlas de vulnerabilidad urbana de España, 2012).

All parents or tutors signed the informed consent form approved by the Research Ethical Committee (No. 2010/41221/I) of the IMIM‐Parc de Salut MAR, Barcelona, Spain, and the FP7‐ERC‐2010‐AdG Ethics Review Committee (268479‐22022011).

### Copper exposure

Each school was measured twice during 1‐week periods separated by 6 months, in the warm (year 2012) and cold (year 2012/2013) periods. Indoor air in a single classroom and outdoor air in the courtyard were measured simultaneously. Several pollutants were measured during class time (Amato et al. 2014; Rivas et al. 2014; Sunyer et al. 2015). Copper specifically was measured during 8 h (09:00–17:00 h) in particulate matter with an aerodynamic diameter <2.5 *μ*m (PM2.5) collected on filters with high‐volume samplers (MCV SA, Spain). After acid digestion of the filter, copper concentrations were determined via ICP‐MS (inductively coupled plasma mass spectrometry). Yearly school air pollution levels were obtained by averaging the two 1‐week measurements. This study was focused on outdoor (courtyard) copper measurements, as they are more directly related to urban pollution, which is the primary interest of this project. All children had been in the school for more than 18 months (and 98% more than 2 years) at imaging assessment, which was carried out after the pollution measurement campaigns.

### Behavioral measurements

Behavioral assessment was focused on testing speed and consistency of motor responses. We used the computerized “Attentional Network Test,” child version (Child ANT) (Rueda et al. 2004), which was developed to specifically assess children's motor speed during attentional challenge. Among the ANT measurements, we used the overall “reaction time” to measure speed and “reaction time standard deviation” to measure trial‐to‐trial consistency of motor responses. A higher reaction time standard deviation indicates higher reaction time variability, which increases with reduced executive and attentional resources depending on the integrity of frontal‐basal ganglia circuits (Bellgrove et al. 2004; MacDonald et al. 2006; Langner and Eickhoff 2013). Normal children become efficient in sustained motor responses during the preadolescent age period contemplated in our study (MacDonald et al. 2006).

The procedure used to administer the task is fully described in a previous report (Forns et al. 2014). The Child ANT takes approximately 20 min to complete. Groups of 10–20 children were assessed together wearing ear protectors and were supervised by one trained examiner per 3–4 children. The task required to respond to a target (yellow‐colored fishes) presented on a computer screen by pressing a key. The whole task contains cues to test different attentional aspects, but outcome measurements for the current study were limited to median reaction time and standard deviation expressed in milliseconds from the correct responses obtained across the 128 trials of the task (four separate blocks of 32 trials each). Commission and omission errors were also registered. Cases with more than 30% commission or omission errors were excluded from further analyses (total nine cases in the whole sample and two cases in the MRI sample).

### Contextual behavioral assessment

Sociodemographic factors were measured using a neighborhood socioeconomic status vulnerability index (based on the level of education, unemployment, and occupation at the census tract) (Atlas de Vulnerabilidad Urbana de España, 2012; http://www.fomento.gob.es/. . ./AtlasVulnerabilidadUrbana/)) according to both school and home address. Parental education was registered for both parents using a 5‐point scale (1 illiterate/2 less than/3 primary/4 secondary/5 university). Standard measurements of height and weight were performed to define overweight and obesity (de Onis et al. 2009). Parents completed the SDQ (Strengths and Difficulties Questionnaire) on child behavioral problems (Goodman 2001). A “difficulties” score ranging from 0 to 40 was generated. Overall school achievement was rated by teachers using a 5‐point scale (from the worse = 1 to the best = 5).

### MRI acquisition

A 1.5‐Tesla Signa Excite system (General Electric, Milwaukee, WI) equipped with an eight‐channel phased‐array head coil and single‐shot EPI (echoplanar imaging) software was used. The imaging protocol included high‐resolution T1‐weighted 3D anatomical images, DTI, and a functional MRI sequence acquired in the resting state.

High‐resolution 3D anatomical images were obtained using an axial T1‐weighted three‐dimensional fast spoiled gradient inversion recovery prepared sequence. A total of 134 contiguous slices were acquired with repetition time 11.9 msec, echo time 4.2 msec, flip angle 15°, field of view 30 cm, 256 × 256 pixel matrix, and slice thickness 1.2 mm.

Diffusion tensor imaging was obtained using spin‐echo single‐shot echo‐planar sequences of 25 directions with a B‐factor of 1000 sec/mm^2^. Twenty‐six slices were acquired with repetition time 8300 msec, echo time 94 msec, thickness 5 mm, no gap, pulse angle 90°, field of view 26 cm, and 128 × 128 acquisition matrix reconstructed into a 256 × 256 matrix.

The functional MRI sequence consisted of gradient recalled acquisition in the steady state with repetition time 2000 msec, echo time 50 msec, pulse angle 90°, field of view 24 cm, 64 × 64‐pixel matrix, and slice thickness 4 mm (interslice gap, 1.5 mm). Twenty‐two interleaved slices were prescribed parallel to the anterior–posterior commissure line covering the whole brain. A 6‐min continuous resting‐state scan was acquired for each participant. Children were instructed to relax, stay awake, and lie still without moving, while keeping their eyes closed throughout. This scan generated 180 whole‐brain EPI volumes. The first four (additional) images in each run were discarded to allow magnetization to reach equilibrium.

### Image preprocessing

#### Anatomical 3D

All the anatomical images were visually inspected before analysis by a trained operator to detect any motion effect. A total of 10 children were discarded as a result of poor quality images and thus the final sample for the 3D anatomical analysis included 253 children. Anatomical 3D data were processed in two separate analyses assessing different anatomical characteristics.

Gray and white matter tissue concentration and volume at a voxel level were measured using Statistical Parametric Mapping (SPM8) (http://www.fil.ion.ucl.ac.uk/spm, Wellcome Department of Cognitive Neurology, London, UK, 2008). SPM VBM (voxel‐based morphometry) algorithms with DARTEL registration were used with the following processing steps: segmentation of anatomical images into gray and white matter tissue probability maps in their native space; estimation of the deformations that best align the images together by iteratively registering the segmented images with their average; finally, generating spatially normalized and smoothed segmentations (5 × 5 × 5 FWHM) using the deformations estimated in the previous step. The analyses were performed with scaling by Jacobian determinants (estimates of volume change during the normalization) to consider tissue volume and without Jacobian scaling to assess the relative concentration of gray matter and white matter. Normalized images were finally transformed to the standard SPM template, resliced to 1.5 mm resolution in MNI (Montreal Neurological Institute) space.

Cortical thickness measurements across the whole cortex were obtained using FREESURFER tools (http://surfer.nmr.mgh.harvard.edu/). Processing steps included removal of nonbrain tissue, segmentation of the subcortical white matter and deep gray matter volumetric structures, tessellation of the gray and white matter boundary, registration to a spherical atlas which is based on the individual cortical folding patterns to match cortical geometry across subjects, and creation of a variety of surface‐based data. Cortical thickness is calculated as the closest distance from the gray/white boundary to the gray/CSF boundary at each vertex on the tessellated surface (Fischl et al. 1999).

#### Diffusion tensor imaging

Diffusion tensor imaging was processed using FMRIB (functional MRI of the brain) Software Library 5.0 (FSL), developed by the Analysis Group at the Oxford Centre for FMRIB (Smith et al. 2004). Diffusion‐weighted images were corrected for motion and eddy current distortions (“Eddy Current Correction” option in the FMRIB Diffusion Toolbox [FDT] version 2.0 in FSL), and a whole‐brain mask was applied using the FSL Brain Extracting Tool. A further rigorous image quality control was carried out to identify potential residual effects of head motion, which involved the visual inspection of each DTI slice for all 25 DTI volumes in all participants. Volumes with slices with signal loss (greater than ~ 10%) or residual artifacts were identified by an expert researcher. DTI full examinations showing one or more degraded images in more than five volumes were discarded. A total of 76 children were removed from the DTI analysis on the basis of this criterion (in addition to 10 cases showing gross image degradation). The final DTI sample involved 177 children showing a mean ± SD of 23.5 (94%) ± 1.9 optimal quality volumes. Subsequently, we estimated FA (fractional anisotropy) maps using FDT in FSL after local fitting of the diffusion tensor model at each voxel (“dtifit”). Next, diffusion data were processed using tract‐based spatial statistics (Smith et al. 2006). Each FA dataset was resliced to a 1 mm × 1 mm × 1 mm anatomical resolution and normalized to standard MNI space via the FMRIB58_FA template using the FMRIB's nonlinear registration tool.

To complement the analysis and assist interpretation of the FA results, we carried out a directional analysis of water diffusion along each (*x*,* y*,* z*) axis separately. We used the diffusion tensor estimated by FSL “dtifit” algorithm to determine diffusion strengths. Geometrically, the procedure involved determining the radius of the arbitrarily oriented ellipsoid along the spatially fixed *x*,* y*, and *z* axes using basic 3D quadratic geometry. As the eigenvectors and eigenvalues were previously normalized to MNI space in all subjects, we were able to carry out group‐level voxel‐wise analyses.

#### Functional MRI

Preprocessing was carried out using SPM8 and involved motion correction, spatial normalization, and smoothing using a Gaussian filter (full‐width half‐maximum, 8 mm). Data were normalized to the standard SPM‐EPI template and resliced to 2‐mm isotropic resolution in MNI space.

The following procedures were adopted to control for potential head motion effects: (1) Conventional SPM time‐series alignment to the first image volume in each subject. (2) Exclusion of 24 children with large head motion (boxplot‐defined outliers) (Pujol et al. 2014b). The finally analyzed sample therefore included 239 children. (3) Both motion‐related regressors and estimates of global brain signal fluctuations were included as confounding variables in first‐level (single‐subject) analyses. (4) Within‐subject, censoring‐based MRI signal artifact removal (Power et al. 2014) (scrubbing) was used to discard motion‐affected volumes. For each subject, interframe motion measurements (Pujol et al. 2014b) served as an index of data quality to flag volumes of suspect quality across the run. At points with interframe motion >0.2 mm, that corresponding volume, the immediately preceding and the succeeding two volumes were discarded. Using this procedure, a mean ± SD of 11.2 (6.2%) ± 13.8 volumes of the 180 fMRI sequence volumes were removed in the analyzed sample. (5) Potential motion effects were further removed using a summary measurement for each participant (mean interframe motion across the fMRI run) as a regressor in the second‐level (group) analyses in SPM (Pujol et al. 2014b).

Functional connectivity maps were generated using procedures detailed in previous reports (Harrison et al. 2013; Pujol et al. 2014a). Maps representative of frontal lobe functional connectivity were obtained by locating seed regions at the medial‐dorsal, lateral (right and left), and medial‐anterior aspects of the frontal cortex using coordinates taken from previous literature (Fox et al. 2005) converted to MNI: medial‐dorsal (*x* = −2, *y* = −2, *z* = 55), right lateral (*x* = 45, *y* = 3, *z* = 15), left lateral (*x* = −45, *y* = 5, *z* = 9), and medial‐anterior (*x* = 1, *y* = 54, *z* = 26). Additional maps were generated from the results obtained in the structural (anatomical and DTI) analyses. The seed regions were centered at the left caudate nucleus at three anterior–posterior levels covering the part of the head of the caudate nucleus showing significant copper effects on its structure (MNI coordinates [*x* = −12, *y* = 20, *z* = 4], [*x* = −12, *y* = 14, *z* = 9], and [*x* = −12, *y* = 8, *z* = 12]).

For each of the striatal locations, the seed region was defined as a 3.5‐mm radial sphere (sampling ~ 25 voxels in 2 mm isotropic space). This was performed using MarsBaR ROI (region of interest) toolbox in MNI stereotaxic space (Brett et al. 2003). Signals of interest were then extracted for each seed region, respectively, by calculating the mean ROI value at each time point across the time series. To generate the seed maps, the signal time course of a selected seed region was used as a regressor to be correlated with the signal time course of every voxel in the brain in order to generate first‐level (single‐subject) voxel‐wise statistical parametric maps (contrast images). The maps were estimated for each seed separately. A high‐pass filter set at 128 sec was used to remove low‐frequency drifts below ~0.008 Hz. In addition, we derived estimates of white matter, CSF, and global brain signal fluctuations to include in the regression analyses as nuisance variables.

### Statistical analysis

A multiple linear regression was used to estimate the source of copper and the relative contributions were given as standardized *β* values. A linear regression was used to estimate the association of copper measurements with motor performance. *β* values are reported as time increments (msec) for each copper measurement unit (ng/m^3^).

Imaging data were analyzed using SPM. Individual anatomical (Jacobian‐modulated and nonmodulated white and gray matter and cortical thickness), DTI, and functional connectivity maps were included in second‐level (group) analyses to map voxel‐wise the correlation across‐subjects between individual brain measurements and individual copper exposure (the measurements obtained in the school of each participant). Results were considered significant with clusters of 1.032 mL (e.g., 129 voxels with a resolution of 2 × 2 × 2 mm) at a height threshold of *P* < 0.005, which satisfied the FWE (family‐wise error) rate correction of *P*_FWE_ < 0.05 according to recent Monte Carlo simulations (Pujol et al. 2014c). Maps in figures are displayed at *t* > 2.3.

## Results

### Copper as an air pollutant

Copper measured in fine particles (PM_2.5_) from school playgrounds showed a mean of 8.7 ng/m^3^ (SD, 3.0; range 3.7–13.8). According to the correlation with specific tracers, the main source of copper was road traffic, but a significant contribution was the result of industrial activity. An additional third source was identified in relation to the close proximity (mean ± SD, 90 ± 58 m) of seven schools to busy overhead‐wire railway lines. For instance, a multiple regression accounted for 70% variance of copper measurements including elemental carbon as a single road traffic tracer (Amato et al. 2014) (standardized *β *= 0.66, *P* < 0.00001), zinc as a single industry tracer (Amato et al. 2014) (standardized *β *= 0.31, *P* = 0.002), and train proximity (standardized *β *= 0.20, *P* = 0.037).

### Relationship of copper exposure with children's performance

Higher copper exposure was associated with poorer motor performance in children. Although in terms of the whole sample (*n* = 2827 after nine exclusions), the association was significant for motor response speed (reaction time, *β *= 2.2 and *P* = 0.006), the effect was more robust on motor response consistency (reaction time standard deviation, *β *= 2.9 and *P* < 0.00001). Such a negative relationship with reaction time variability was significant in the group of children receiving MRI (*n* = 261 after two exclusions; *β *= 4.2 and *P* = 0.026). Table 1 shows descriptive statistics.

### Neuroimaging results

**Figure 1:**
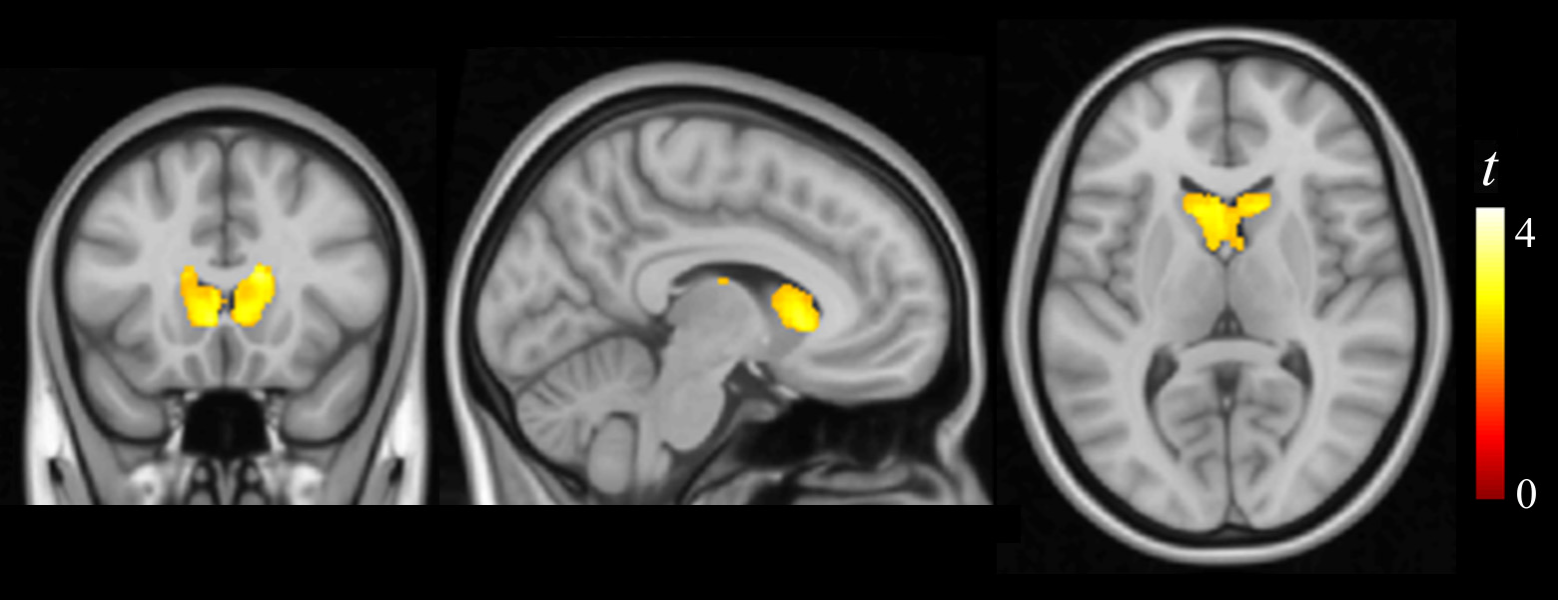
Correlation of copper measurements with brain tissue composition. Higher copper levels correlated with higher gray matter (presumably expressing lower white matter) concentration in the caudate nucleus bilaterally. The right hemisphere corresponds to the right side of axial and coronal views.

Three‐dimensional anatomical (T1‐weighted) MRI and DTI were used to assess the potential association of copper exposure with alteration in the fine brain structure. Copper was associated with higher gray matter concentration (i.e., a higher proportion of gray matter in the tissue) in the striatum, specifically in the caudate nucleus (Fig. 1 and Table 2), with no effect on tissue volume. This finding potentially expresses a relative reduction of striatum white matter (i.e., of white matter “striae” that actually give the corpus striatum its anatomical name). No other significant alterations were identified with 3D anatomical MRI with the exception of an area of increased cortical thickness in the supplementary motor area of the left hemisphere (data not shown).

**Table 2:** MRI correlation results

	Cluster size, mL	*x*,* y*,* z*	*t*
Copper versus gray matter concentration			
R Caudate nucleus‐positive correlation	4.09	8, 19, 3	3.12
L Caudate nucleus‐positive correlation	4.09 (same cluster)	−3, 16, 6	3.12
Copper versus fractional anisotropy			
R Caudate nucleus‐positive correlation	1.40	11, 9, 9	3.8
L Caudate nucleus‐positive correlation	9.23	−8, 9, 10	3.9
L Supracaudate white matter‐positive correlation	9.23 (same cluster)	−17, 20, 25	3.6
L Suprathalamic white matter‐positive correlation	9.23 (same cluster)	−20, −17, 28	3.4
R Corpus callosum‐positive correlation	1.90	17, −40, 13	3.8
Reaction time versus fractional anisotropy			
L Supracaudate white matter‐positive correlation	1.46	−15, 11, 21	3.6
Reaction time SD versus fractional anisotropy			
L Supracaudate white matter‐positive correlation	4.29	−17, 10, 19	3.9
R Supracaudate white matter‐positive correlation	3.60	19, 15, 20	4.1
*Copper versus functional connectivity*			
Left caudate nucleus seed map			
R Frontal operculum‐negative correlation	5.85	48, 2, 16	4.2
L Frontal operculum‐negative correlation	3.30	−38, 14, 12	3.9
Right frontal operculum seed map			
L Caudate nucleus‐negative correlation	2.35	−14, 22, 8	3.9
R Caudate nucleus‐negative correlation	1.39	16, 22, 10	3.1
Left frontal operculum seed map			
L Caudate nucleus‐negative correlation	1.30	−16, 24, 6	4.4
Frontal medial seed map			
L Frontal operculum‐positive correlation	3.29	−44, 32, −2	3.2
L Auditory cortex‐positive correlation	1.72	−50, −18, 2	3.5
L Medial frontal cortex‐positive correlation	1.42	−12, 22, 44	3.9
R Visual cortex‐negative correlation	12.81	14, −54, 2	3.8
Supplementary motor area seed map			
L Supramarginal gyrus‐positive correlation	1.08	−56, −34, 24	3.6

SD, standard deviation.

*x*,* y*,* z* coordinates given in MNI (Montreal Neurological Institute) space. Statistics at corrected threshold *P*_FWE_ < 0.05 estimated using Monte Carlo simulations.

**Figure 2:**
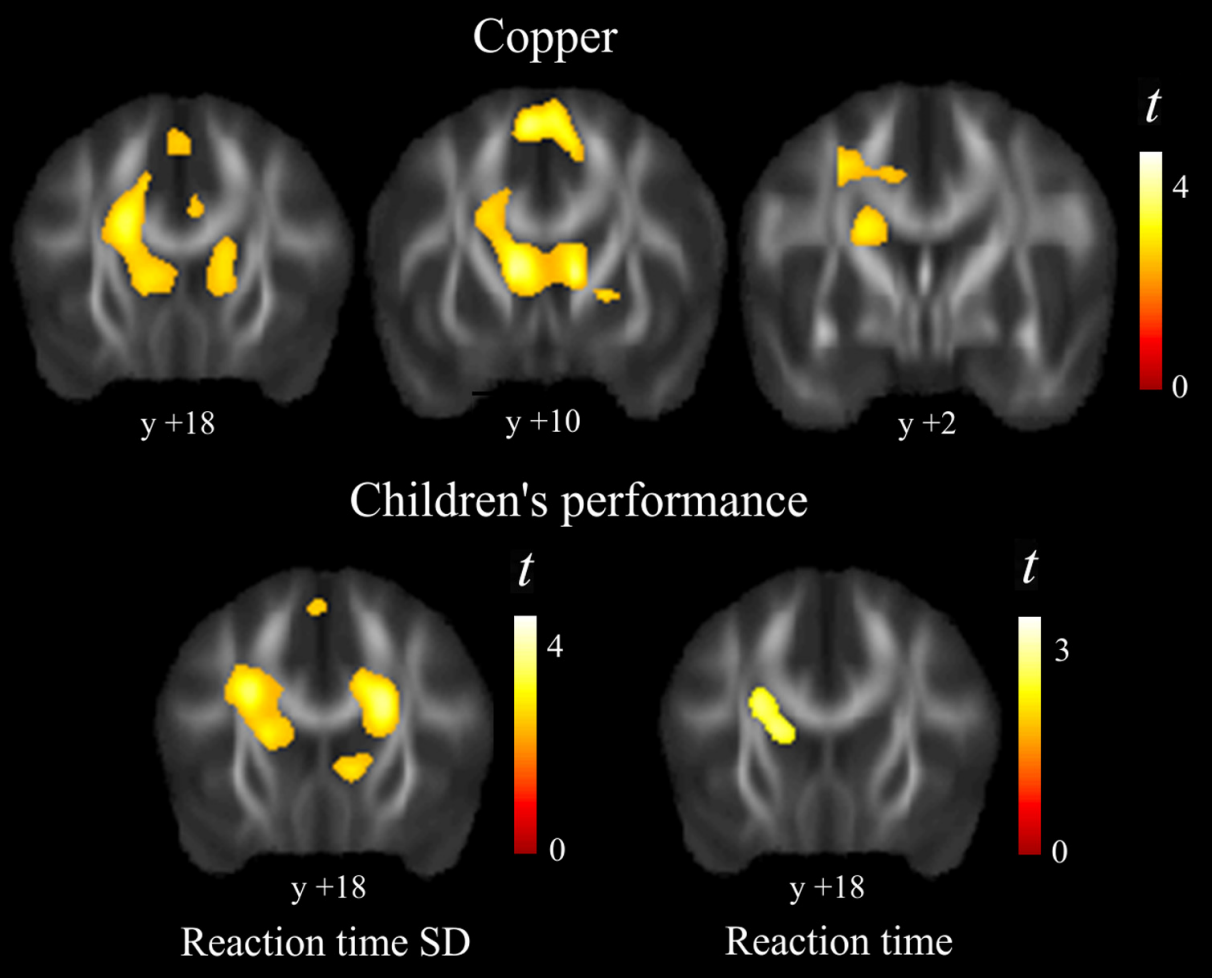
Diffusion tensor imaging results. Higher copper levels correlated with higher FA (fractional anisotropy) predominantly in caudate nucleus region (top panel). The correlation with motor performance (bottom panel) showed both slower reaction time and larger reaction time standard deviation associated with higher FA in white matter adjacent to the caudate nucleus. The right hemisphere corresponds to the right side. *Y* denotes “*y*” MNI coordinates.

**Figure 3:**
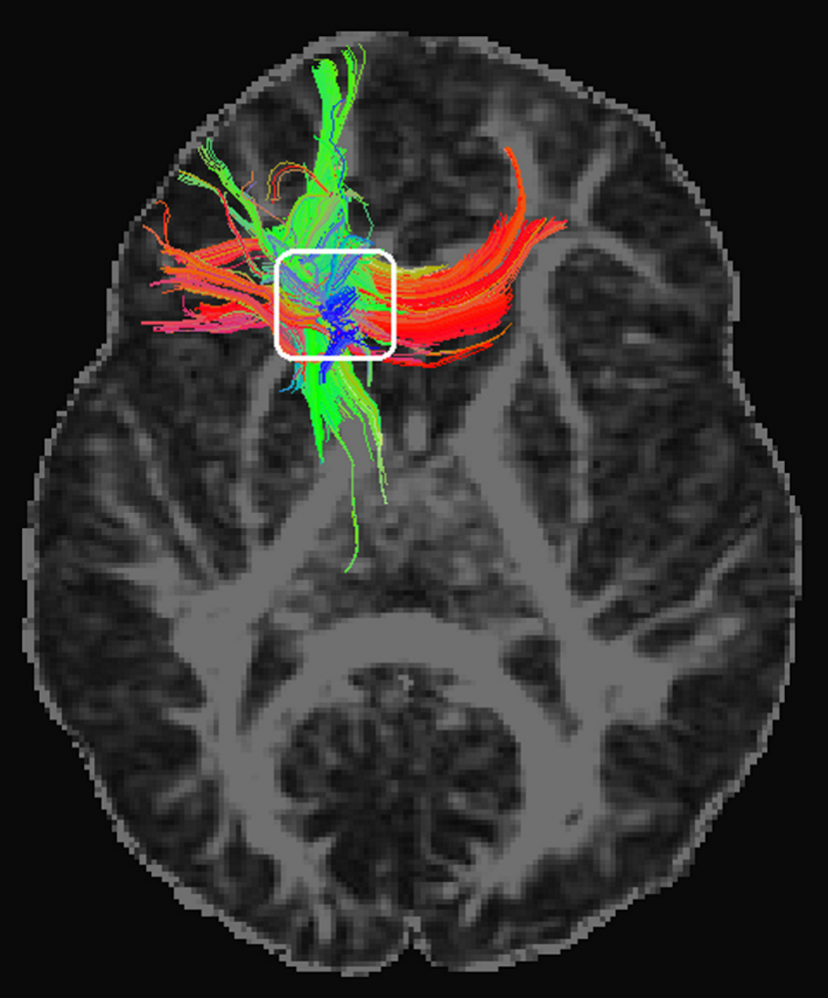
Diffusion tensor imaging tractographic display of a representative participant. The colors are coded to show diffusion‐defined left–right tracts in red, anterior–posterior tracts in green, and superior–inferior tracts in blue. Note convergence of the three directions in our region of interest (white rectangle).

Results from the DTI analysis were highly consistent with the anatomical results (Fig. 2, Table 2). Copper was associated with an increase of neural tissue FA. This DTI measurement may express the extent to which an anatomical structure is composed of white matter tracts showing one dominant direction. In brain regions containing tracts with a single direction, FA increases as a result of brain maturation. Nevertheless, in complex structures with tracts crossing in different directions, higher FA may denote less mature or less structured tissue (Douaud et al. 2011; Jones et al. 2013). In our analysis, higher copper levels were associated with higher FA in white matter close to the caudate nucleus and in the caudate nucleus itself. This region is anatomically characterized as showing superior–inferior, posterior–anterior, and lateral–medial crossing white matter tracts (see Fig. 3 and Kotz et al. 2013). Within this scenario, a less mature structure will show higher FA (the effect of copper in our study).

A voxel‐wise correlation analysis with behavior measurements helped to establish the nature of the identified changes associated with copper exposure. Reaction time and, mostly, reaction time variability showed significant positive correlation with FA of white matter adjacent to the caudate nucleus (Fig. 2). Thus, in the direction of copper exposure findings, slower children and the children with less consistent motor response exhibited higher FA in this region.

**Figure 4:**
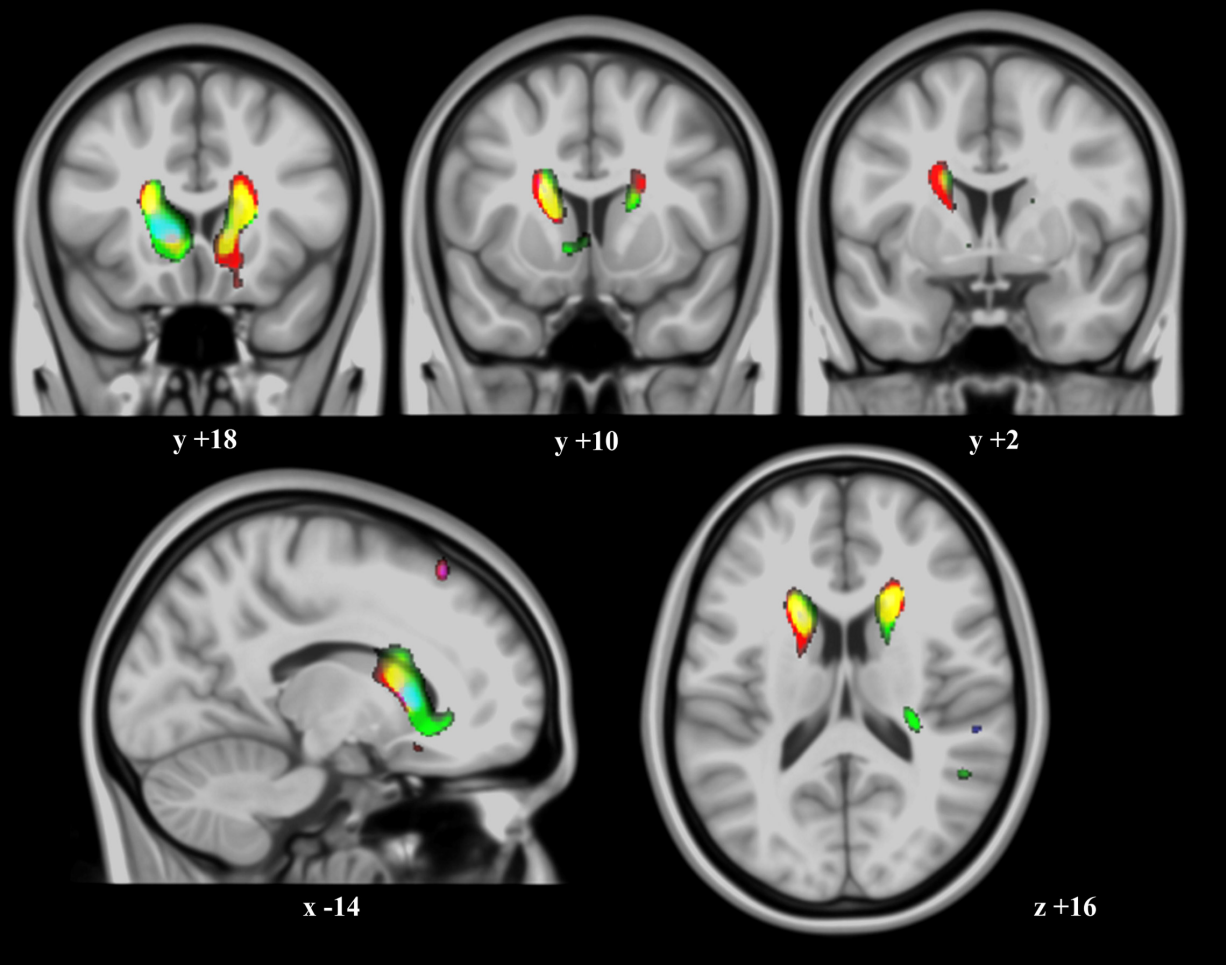
Water diffusion direction changes related to copper. Correlation of copper measurements with water diffusion along the three (*x*,* y*,* z*) axes separately and superimposed in a single RGB color display (thresholded at *P* = 0.001 in MRIcron^®^). Color regions express sites where higher copper levels were associated with reduced diffusion for one or more directions. The most evident changes were along the right–left (red), anterior–posterior (green) directions, or both (yellow) (note that regions with mixed effects show RGB composite colors). The right hemisphere corresponds to the right side of axial and coronal views. *X*,* Y*,* Z*, denote MNI coordinates.

To investigate copper effects on the neural track architecture in the caudate nucleus region, we analyzed tissue diffusion along the three orthogonal (*x*,* y*,* z*) directions separately. Higher copper exposure was associated with a complex combination of diffusion changes in the caudate nucleus region, with distinct sectors showing reduced diffusion in one or more directions (Fig. 4). In other words, copper was related to changes in the complex architecture of neural tissue diffusion, further supporting the notion that white matter pathways in the caudate nucleus region may be affected by copper exposure.

**Figure 5:**
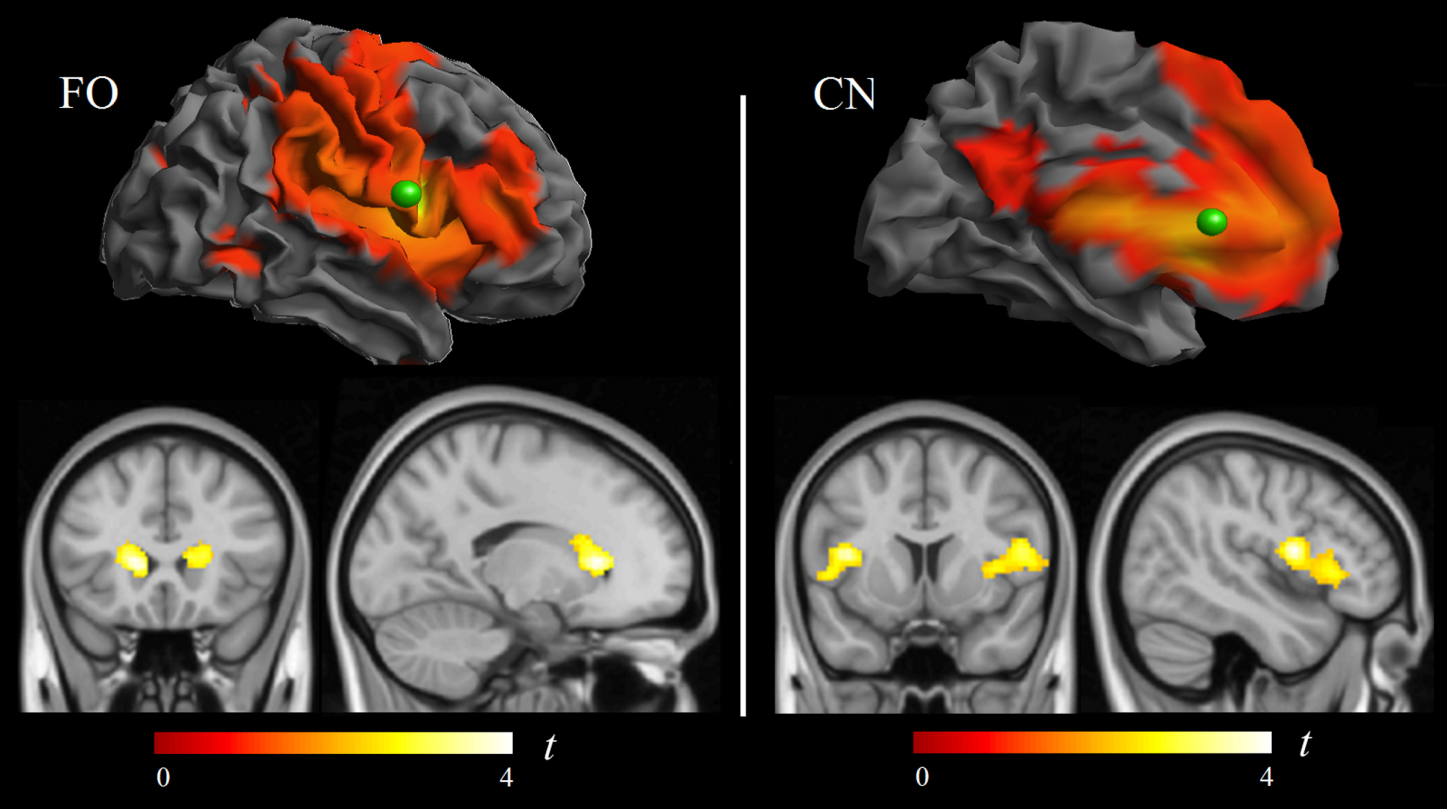
Correlation of copper measurements with functional connectivity MRI. Within the frontal operculum functional connectivity map (left panel), copper was associated with a connectivity reduction between the frontal (seed) region of interest (green sphere) and the caudate nuclei. Reciprocally, within the caudate nucleus functional connectivity map (right panel), copper was associated with a connectivity reduction between the caudate nucleus seed region (green sphere) and the frontal lobe opercula.

The functional significance of the identified basal ganglia changes was further investigated by assessing functional connectivity in the basal ganglia network. Functional connectivity MRI maps representative of frontal‐basal ganglia circuits were generated using coordinates taken from previous works and from the current anatomical and DTI results centered at the caudate nucleus (see Methods). The most relevant finding was the association of higher copper exposure to a reciprocal reduction of functional connectivity between the caudate nucleus and the frontal lobe operculum bilaterally (Fig. 5). This association was consistent with the anatomical and DTI results and notably specific in terms of functional anatomy. Remarkably, reduced caudate‐to‐frontal operculum connectivity was the only significant finding in three functional connectivity maps (see Table 2).

The effect of potential confounders was tested for each significant finding including age, sex, academic achievement, academic difficulties score, obesity, parental education, home and school vulnerability index, and public/nonpublic school category as covariates. No single confounder or combination showed a significant effect. That is, decreases in *β* estimates after the inclusion of confounders in a regression model were very small with no variables affecting the primary results with *β* reductions >10%. Finally, potential alterations associated with Mn (manganese) were also investigated, as this element may be associated with basal ganglia alterations. We did not, however, find any significant finding associated with manganese. Moreover, the effect of copper had a tendency to be more robust when adjusted by Mn (e.g., the primary correlation between copper and motor response consistency showing *β *= 2.9 in the whole sample, showed *β *= 3.3 after entering Mn measurements as a covariate). Similarly, the effect of copper remained (no *β* decrease > 10%) after additionally adjusting by other representative elements (Table 3, i.e., C, Pb, Fe, and Sb).

**Table 3:** Potentially relevant airborne elements and their correlation with Cu

Measures from 39 schools	Mean ± SD	Range	Corr, with Cu *r* values	Shared variance (adjusted *r* ^2^ × 100)
Carbon (C), *μ*g/m^3^	1.5 ± 0.7	0.6–3.9	0.41	15%
Manganese (Mn), ng/m^3^	15.3 ± 13.8	3.7–64.8	0.22	2%
Lead (Pb), ng/m^3^	8.1 ± 2.8	4.3–16.8	0.46	19%
Iron (Fe), *μ*g/m^3^	0.6 ± 0.6	0.1–3.0	0.15	0.4%
Antimony (Sb), ng/m^3^	1.1 ± 0.4	0.4–2.4	0.75	55%

Cu, copper; SD, standard deviation.

## Discussion

Airborne copper was significantly associated with poorer motor performance and detectable brain damage in developing children. The alterations are highly consistent with the known consequences of copper excess on the brain with basal ganglia as the main target. The associations were demonstrated with copper levels common in urban environments, thus suggesting a risk to large populations with potentially significant implications for public health. Children may be particularly vulnerable to copper as an agent capable of interfering with brain development during critical developmental stages. Consistent with our results, a recent study has reported a significant association between high copper levels in blood and poorer cognitive performance in normal school children (Zhou et al. 2015).

The *β* weights reported in Table 4 may help in determining the biological relevance of behavior and imaging changes associated with air copper in our study. While the change related to reaction time was small, the effect on motor response consistency (measured as reaction time standard deviation) was more important. For instance, the increase in one unit (ng/m^3^) of air copper predicts an increase of 2.9 msec in reaction time standard deviation. Therefore, if the range of copper measurements is 10 units, from the least (3.7 ng/m^3^) to the most polluted school (13.8 ng/m^3^), the potential variation predicted is 29 msec, which is approximately one third of the standard deviation of this motor performance measurement (SD, 91 msec). The magnitude of the effect on the caudate nucleus structure and function was of the same order. Overall, our conclusion is that the effect of copper is subtle, but biologically meaningful.

**Table 4:** Behavior and imaging changes associated with air copper (copper range, 3.7–13.8 ng/m^3^)

Whole sample (*n* = 2827)	Mean ± SD	*β* Coefficient (adjusted *β*)^b^	95% CI (adjusted 95% CI)	*t*	*P*
Motor speed (reaction time)	671 ± 124 msec	2.2 (4.7) ms/(ng/m^3^)	0.6 to 3.7 (1.8 to 7.5) ms/(ng/m^3^)	2.7	0.006
Motor response consistency (reaction time SD)	235 ± 91 msec	2.9 (3.4) ms/(ng/m^3^)	1.7 to 4.0 (1.4 to 5.5) ms/(ng/m^3^)	4.9	8e‐7
MRI sample (*n* = 261)^a^					
Motor response consistency (reaction time SD)	224 ± 91 msec	4.2 (9.6) ms/(ng/m^3^)	0.5 to 7.9 (1.7 to 17.6) ms/(ng/m^3^)	2.2	0.026
Gray matter concentration L caudate nucleus	14.2 ± 3.9 GMc	0.3 (0.3) GMc/(ng/m^3^)	0.1 to 0.4 (0.1 to 0.5) GMc/(ng/m^3^)	3.1	0.001
Fractional anisotropy DTI L caudate nucleus	16 ± 1 FAi	0.1 (0.1) FAi/(ng/m^3^)	0.06 to 0.2 (0.05 to 0.2) FAi/(ng/m^3^)	3.9	0.0001
Functional connectivity L frontal cortex to L caudate	0.3 ± 1.1 FCi	−0.1 (−0.1) FCi/(ng/m^3^)	−0.14 to −0.05 (−0.2 to −0.1) FCi/(ng/m^3^)	−4.4	0.00001

SD, standard deviation; *β*,* β* coefficients from the regressions with copper as the predictor factor; GMc, percentage of gray matter concentration; FAi, anisotropy index expressed in the range of 0–100 units; FCi, strength of functional connectivity expressed in arbitrary units.

a *n* varied in each imaging modality (see Methods section).

b Socioeconomic status, a general indicator of traffic pollution (elemental carbon) and other potentially toxic agents (Pb, Mn, Sb, and Fe) were used in the adjusted model.

In the current study, we have identified copper as one road traffic‐related pollutant. Traffic‐related copper is thought to be mostly released from brake pads (Hulskotte et al. 2007) and it was the main source in our study. A significant proportion of copper, however, comes from industrial activity and a third source seems to be the result of railway traffic. This is consistent with reports indicating that the air in busy train stations contains large amounts of copper generated by overhead train wire supplying electrical power (Kim et al. 2010; Loxham et al. 2013).

Although a normal oral diet contains a considerable amount (~1 mg) of copper (Morris et al. 2006), our data suggest that relatively lower levels are neurotoxic in chronic airborne exposures. Ingested copper is mostly incorporated into ceruloplasmin (safe copper) and any excess is removed by excretion into the bile (Madsen and Gitlin 2007). There is evidence indicating that the “toxic copper” is actually the circulating free (i.e., nonceruloplasmin bound) copper (Squitti 2014c; Pal and Prasad 2015), which is the only fraction capable of penetrating the brain parenchyma (Zheng and Monnot 2012). Inhaled copper may notably circumvent the liver regulation and safe binding to ceruloplasmin. So, nonceruloplasmin‐bound copper absorbed in the respiratory tract can more easily enter the brain and achieve higher tissue concentrations.

Environmental copper has been proposed as a risk factor for neurodegenerative disease (Morris et al. 2006; Caudle 2015; Pal and Prasad 2015). The highest exposures occur through the inhalation of fumes generated from welding, as well as metal mining and smelting activities. Long‐term (20 years) occupational copper exposure has been shown associated with 2.5‐fold increase in risk for Parkinson's disease (Gorell et al. 2004; Caudle 2015) and with a younger age at onset (46 years) (Racette et al. 2001). However, manifest neurological disease seems not to be a necessary outcome in occupational copper exposure, which suggests different susceptibilities among individuals and the potential interaction between a genetic predisposition and copper availability. This is obvious in the paradigmatic Wilson disease where a similar genetic alteration may correspond to a range of clinical severity and, on the other hand, the neurological disorder may be not expressed if copper intake is properly controlled (Bandmann et al. 2015). Also, there is a possibility that the toxic capacity of environmental copper excess saturates at relatively lower copper levels (i.e., high environmental copper concentrations and very high concentrations could generate similar brain damage if the assumption is correct).

The neurotoxic action of copper via oxidative stress and mitochondrial injury (Eskici and Axelse 2012; Bandmann et al. 2015) may also depend on tissue energy consumption rates. In children, the highly energetic basal ganglia appear to be the main target. This occurs in our study and in Wilson's disease, in which the lenticular nucleus (putamen and globus pallidus) was originally described with the most dramatic pathological changes (Compston 2009), although the caudate nucleus may show the most severe reduction in glucose consumption (Hermann 2014). By contrast, a more persistent subtle excess of copper throughout life could be responsible for protracted but widespread brain damage. In this context, an increasing amount of evidence indicates that copper may play a causal role in late‐onset neurodegenerative disorders (Bush 2003; Bandmann et al. 2015). For example, nonceruloplasmin‐bound copper levels are higher than normal reference values in up to 60% of Alzheimer's disease patients (Squitti 2014c; Squitti et al. 2014b). Also, high‐nonceruloplasmin‐bound copper concentrations were associated with an increased rate of mild cognitive impairment conversion to full Alzheimer's disease (Squitti et al. 2014a). A prospective study revealed an association between a diet simultaneously high in copper and saturated fats and cognitive decline (Morris et al. 2006). In a variety of experimental animal studies, oral copper intake resulted in significant amyloid *β* deposition and performance decline (Alzheimer's disease‐like pathology), even at low concentrations (Pal and Prasad 2015).

In the broader context of traffic‐related pollution, the effect of air pollutants may be more dramatic when the exposure involves earlier developmental periods. Indeed, Peterson et al. (2015) have provided evidence of brain structural alterations in later childhood associated with prenatal pollutant exposure affecting large areas of the left‐hemisphere white matter, and a less severe effect associated with postnatal exposures at age 5 years. On the other hand, recent studies have revealed that long‐term ambient air pollution exposure may ultimately affect brain tissue volume in older people (Chen et al. 2015; Wilker et al. 2015).

A general limitation when assessing children with MRI is the potential effect of head movements on image quality, particularly on functional MRI and DTI acquisitions. We have considered this issue carefully and adopted several means to rigorously control the effects of motion on functional MRI (see Methods). In the case of DTI, we decided to exclude cases with detectable image degradation, as no correction procedure is wholly efficient. A post hoc analysis on DTI using less rigorous exclusion criteria (*n* = 242) showed similar but weakened DTI results, indicating that the data obtained in the more selective sample (*n* = 177) were most probably not the result of motion effects. Also, a higher MRI signal may be obtained using a higher magnetic field (i.e., 3‐Tesla magnets). Although we did have the 3‐Tesla option, the present study was developed using a 1.5‐Tesla magnet following the recommendations of the FP7‐ERC Ethics Review Committee to limit magnetic field strength in children.

## Conclusions

Our study has revealed that apparently safe school environments may indeed to expose developing children to the harmful effects of air pollutants. Tolerated amounts of airborne copper were associated with poorer motor performance in children in the city of Barcelona. The effect on motor performance was directly associated with changes in the structure and function of the basal ganglia, suggesting underdevelopment of the caudate nucleus complex neural connections.

## Conflict of Interest

We have no competing interests.
